# Pretreatment lactate dehydrogenase may predict outcome of advanced non small‐cell lung cancer patients treated with immune checkpoint inhibitors: A meta‐analysis

**DOI:** 10.1002/cam4.2024

**Published:** 2019-03-07

**Authors:** Zhibo Zhang, Ye Li, Xiang Yan, Qi Song, Guoqiang Wang, Yi Hu, Shunchang Jiao, Jinliang Wang

**Affiliations:** ^1^ Department of Medical Oncology Chinese PLA General Hospital Beijing China; ^2^ Department of Graduate Administration Chinese PLA General Hospital Beijing China; ^3^ 209 Hospital of Chinese PLA Mudanjiang China; ^4^ The Medical Department 3D Medicines Inc. Shanghai China

**Keywords:** Immunotherapy, Neutrophil-to-lymphocyte ratio, Non-small cell lung cancer, Peripheral blood biomarker

## Abstract

The main aim of this study is to investigate whether baseline lactate dehydrogenase (LDH) is associated with the clinical outcome of non small‐cell lung cancer (NSCLC) patients treated with immune checkpoint inhibitors (ICIs). We searched Pubmed, the Cochrane Central library and Embase for peripheral blood biomarker of LDH in advanced NSCLC patients treated with ICIs. We extracted the hazard ratio (HR) with 95% confidence interval (CI) for the progression free survival (PFS) and overall survival (OS) and performed meta‐analysis of HR. Pooled estimates of treatment outcomes were calculated by stata 15.1. Six studies with 1136 patients were included in this study. The pooled results of univariate analysis suggested that an elevated pretreatment LDH level was correlated with significant shorter PFS (HR = 1.53, 95% CI 1.27‐1.83, *P* < 0.001) and OS (HR = 2.11, 95% CI 1.43‐3.11, *P* < 0.001). The association remained significant in the multivariate analysis that elevated pretreatment LDH level was associated with poor PFS (HR = 1.62, 95% CI 1.26‐2.08, *P* < 0.001) and OS (HR = 2.38, 95% CI 1.37‐4.12, *P* = 0.002). A high pretreatment LDH level was significantly correlated with shorter PFS and OS. Pretreatment LDH may serve as a predictive biomarker for advanced NSCLC patients treated with ICIs.

## 1 INTRODUCTION

Cancer continues to be the most threatening disease to human health.^1^ Lung cancer remains the most frequent cause of cancer related death worldwide and affects over 1.8 million patients per year.^2^ Non small cell lung cancer (NSCLC) accounts for 85% of lung cancer, a majority of which present with advanced metastatic disease and median survival remains below 12 months.^3^

The advent of cancer immunotherapy especially immune checkpoint inhibitors (ICIs), has brought about a shift in the landscape of advanced‐stage cancer treatment,^4^ especially in NSCLC patients. However, the benefits remain limited to a subset of patients. Biomarkers such as PD‐L1 expression, tumor mutational burden (TMB), neoantigen load, tumor‐infiltrating lymphocytes, and immune‐regulatory mRNA expression signatures are potentially applicable to the clinical selection of patients for ICIs; however, the detection of these biomarkers relied on the adequate tumor tissue, which is challenging in clinical setting. Thus, serum biomarkers are urgently needed as they provide a convenient and nearly non invasive evaluation.

Among all the potential serum biomarkers, lactate dehydrogenase (LDH) is a housekeeping enzyme released by rapidly growing tumors that correlates with tumor burden. Recent studies have demonstrated that elevated pretreatment level of LDH is associated with poor outcome in several cancer types and baseline LDH level may predict the prognosis of patients treated with ICIs.^5^, ^6^, ^7^ However, the predictive role of LDH in NSCLC patients treated with ICIs is uncertain. We conduct this meta‐analysis to identify whether baseline LDH level is correlated with the outcome of advanced NSCLC patients treated with ICIs. Our results suggest that a high pretreatment LDH level was significantly correlated with poor survival and a baseline serum LDH may serve as a potential predictive biomarker for NSCLC patients treated with ICIs.

## 2 METHOD

### 2.1 Search strategy

A search for relevant published and unpublished studies was performed using Embase, PubMed, and Cochrane central Library. The search terms utilized were “immune check point inhibitor”, “cytotoxic T lymphocyte antigen‐4”, CTLA‐4, “programmed death‐1 receptor”, “programmed death ligand‐1”, “PD‐1 inhibitor”, “PD‐L1 inhibitor”, ICI, immunotherapy, nivolumab, pembrolizumab, atezolizumab, avelumab, durvalumab, ipilimumab, “lactate dehydrogenase”, LDH, predictor, predict, prognosis, prognostic, lung cancer, “non small‐cell lung cancer”, NSCLC. The last search was updated on June 13, 2018. Both free text and medical sub‐headings (MeSH) terms were used in the search strategy.

### 2.2 Inclusion criteria

The following articles were included in the analysis: (a) Human studies investigated NSCLC patients receiving ICIs treatment; (b) Determination of the relationship between baseline LDH level and prognosis; (c) Hazard ratio (HR) with 95% CI were presented for OS and/or PFS; (d) If the same population was used by two or more studies, only the one with the largest sample size and latest information was included; (e) the full text was available.

### 2.3 Exclusion criteria

The following studies were excluded from the analysis: (a) Case reports, reviews, comments, editorials, letters or articles unrelated with our topics; (b) Publication in a language other than English.

### 2.4 Data extraction

For each included study, we extracted the data including first author's name, the year of publication, district of study, type of immune checkpoint inhibitor, the total number of patients, sex, age, cut‐off value of LDH, histology, study design, and study outcomes. Two researchers (Zhibo Zhang and Ye Li) independently extracted the data of HRs and the associated 95% CIs for PFS and OS outcomes from both univariate and multivariate analyses. Any discrepancy was resolved by discussion. The present review was prepared according to Preferred Reporting Items for Systematic reviews and Meta‐Analyses (PRISMA).

### 2.5 Quality assessment

As previously reported,^8^ two researchers (Zhibo Zhang and Ye Li) independently assessed the quality of the included studies using following criteria: (a) Representativeness of population; (b) Non exposed cohort; (c) Ascertainment of exposure; (d) Outcome not present at start of study; (e) Appropriate confounding measurement and account; (f) Sufficient measurement of outcomes; (g) Completeness of follow‐up. Studies with a score of higher than 7 were considered as high quality and a score of lower than 7 were defined as low quality. Any disagreement was resolved by consensus.

### 2.6 Statistical analysis

We used the method of random‐effects inverse‐variance‐weighted to pool outcomes, which is calculated by HR and its 95% CI to estimate the size of the treatment benefit. We used the *I* ^2^ statistics to detect any heterogeneity between different studies. A result of *P* > 0.1 and *I* ^2^ < 50% indicated that no significant between‐study heterogeneity was present. Publication bias was evaluated by examining the funnel plot of the effect size for each study. We set the nominal level of significance 5% and all 95% CIs were 2‐sided. All statistical analyses were performed using STATA V.15.1 (Stata Corporation, College Station, Texas, USA).

## 3 RESULTS

### 3.1 Selection of eligible studies

We identified 1199 articles after searching online databases. By verifying related terms in the titles and abstracts, 1110 articles did not meet the inclusion criteria, including 292 duplicate records, 151 irrelevant articles, 585 with no usable data, and 82 without full text. With further reading the whole article, we excluded 83 literatures, all of which were reviews or case reports. Finally, 6 studies were selected for the present meta‐analysis.^9^, ^10^, ^11^, ^12^, ^13^, ^14^ Data from all included literatures were obtained from published manuscripts. A flow chart describing the eligible study selection was shown in Figure S1.

### 3.2 Characteristics of included studies and quality assessment

Six studies with 1136 patients were included in our study. Characteristics of the included studies are summarized in Table 1. In summary, all included studies were retrospective, which were published between 2017 and 2018. Of the 6 included studies, 3 were carried out in Japan and the other 3 studies were conducted in Spain, Switzerland, and France, respectively. Regarding the type of ICI used, four studies reported using Nivolumab,^9^, ^11^, ^12^, ^13^ the remaining 2 studies did not specify the type of ICI used.^10^, ^14^ All of the included studies calculated baseline LDH level. The cut‐off values of LDH level were various, and most of them were among the normal range. Five of 6 studies reported PFS, and 4 of 6 studies reported OS. Three of 6 studies reported both PFS and OS. Newcastle Ottawa Scale (NOS) was used to assess the quality of included studies. The results of quality assessment are listed in Table 2. Four studies had a quality score of 7 and 2 studies have a score of 8.

**Table 1:** Characteristics of included studies for the meta‐analysis on prognostic utility of the LDH in NSCLC patients receiving ICIs

Characteristics	Am Martinez De Castro et al^14^	Stefan Diema et al^13^	Junko Tanizaki et al^9^	Taniguchi et al^11^	Kataoka et al^12^	Laura Mezquita, MD et al^10^
Year	2017	2017	2017	2017	2017	2018
District	Spain	Switzerland	Japan	Japan	Japan	France
Sample	94	52	134	201	189	466
Male/female	Not stated	29/23	90/44	135/66	139/50	301/165
Age, years, median (range)	62 (39‐86)	68	Not stated	68 (27‐87)	69 (38‐88)	62 (29‐86)
Squamous carcinoma/adenocarcinoma/others	40/50/4	18/30/4	33/90/11	42/142/17	Not stated	159/270/37
ICI	PD‐1/PD‐L1	Nivolumab	Nivolumab	Nivolumab	Nivolumab	PD‐1/PD‐L1
LDH detection	Baseline	Baseline	Baseline	Baseline	Baseline	Baseline
LDH ULN	400	246	222	240	217	ULN
Clinical outcomes	OS	PFS, OS	PFS, OS	PFS	PFS	PFS, OS
Univariate analysis (LDH ≥ ULN vs LDH < ULN)						
OS	2.22 (1.19‐4.17)	1.05 (0.37‐2.97)	2.21 (1.1‐4.45)	—	—	2.44 (1.47‐4.04)
PFS	—	1.07 (0.46‐2.48)	1.15 (0.75‐1.74)	1.69 (1.19‐2.39)	1.6 (1.15‐2.23)	1.17 (1.16‐2.69)
Multivariable analysis (LDH ≥ ULN vs LDH < ULN)						
OS	—	—	2.05 (0.71‐5.96)	—	—	2.51 (1.32‐4.76)
PFS	—	—	—	1.63 (1.15‐2.31)	1.6 (1.12‐2. 3)	—
Study design	Retrospective cohort	Retrospective cohort	Retrospective cohort	Retrospective cohort	Retrospective cohort	Retrospective cohort

ULN, upper limit of normal; HR, hazard ratio; OS, overall survival; PFS, progression‐free survival; LDH, lactate dehydrogenase.

**Table 2:** Methodological characteristics of included studies and quality score

N0.	Authors	Year	Representativeness of population	Non exposed cohort	Ascertainment of exposure	Outcome not present at start of study	Appropriate confounding measurement and account	Sufficient measurement of outcomes	Completeness of follow‐up	Overall score
1	Am Martinez De Castro et al^14^	2017	0	1	1	1	2	1	1	7
2	Stefan Diema et al^13^	2017	0	1	1	1	2	1	1	7
3	Junko Tanizaki et al^9^	2017	0	1	1	1	2	1	2	8
4	Taniguchi et al^11^	2017	0	1	1	1	2	1	1	7
5	Kataoka et al^12^	2017	0	1	1	1	2	1	1	7
6	Laura Mezquita, MD et al^10^	2018	0	1	1	1	2	1	2	8

### 3.3 Outcomes of included studies

**Figure 1:**
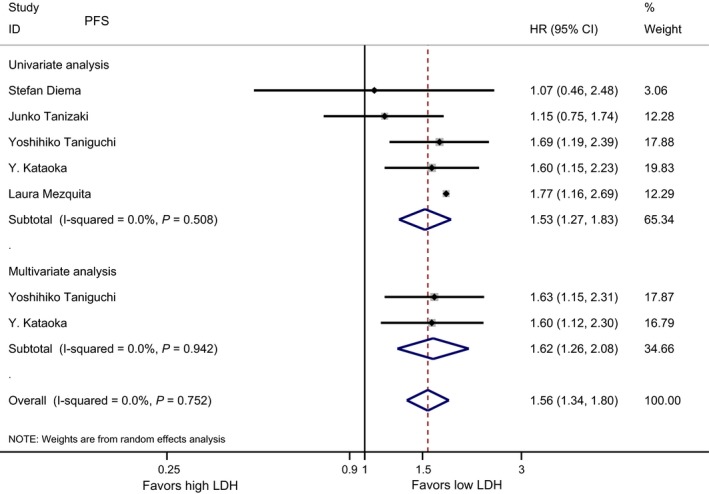
Meta‐analysis of the associations between pretreatment lactate dehydrogenase (LDH) and PFS

Five studies with 1042 cases were included in the final analysis of association between baseline LDH and PFS. As showed in Figure 1, the pooled result suggested that a low baseline LDH level was correlated with significantly longer PFS in the univariate analysis (HR = 1.53, 95% CI 1.27‐1.83, *P* < 0.001), and the pooled results of multivariate analysis revealed that elevated baseline LDH level remained significantly associated with poor PFS (HR = 1.62, 95% CI 1.26‐2.08, *P* < 0.001).

**Figure 2:**
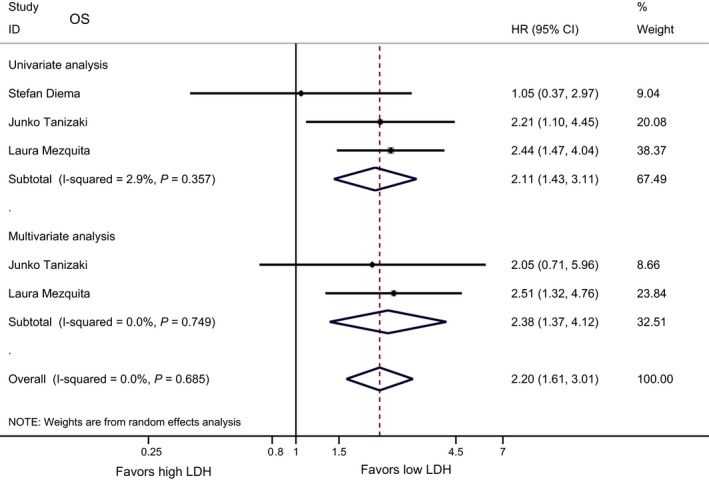
Meta‐analysis of the associations between pretreatment lactate dehydrogenase (LDH) and overall survival (OS)

Four studies with 746 patients were included in the final analysis of association between baseline LDH and OS. In the univariate analysis, the pooled result of suggested that low pretreatment LDH level was correlated with significantly longer OS (HR 2.11, 95% CI 1.43‐3.11, *P* < 0.001), the pooled results of multivariate analysis revealed that elevated baseline LDH level was remained significantly associated with poor OS (HR = 2.38, 95% CI 1.37‐4.12, *P* = 0.002, Figure 2).

### 3.4 Heterogeneity

For PFS, no significant statistical heterogeneity was observed in either univariate or multivariate analyses (*I* ^2^ = 0%, *P* = 0.508; *I* ^2^ = 0%, *P* = 0.942); For OS, we did not observe significant statistical heterogeneity by either univariate or multivariate analysis (*I* ^2^ = 2.9%, *P* = 0.357; *I* ^2^ = 0%, *P* = 0.749).

### 3.5 Publication bias

**Figure 3:**
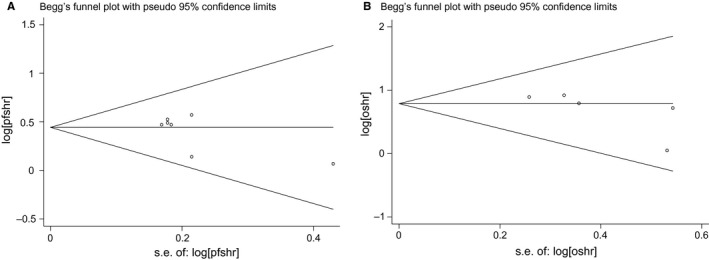
Funnel plot for publication bias in progression free survival (PFS) (A) or overall survival (OS) (B). HR, hazard ratio; SE, standard error

As shown in Figure 3, the funnel plots were almost symmetrical and Egger's test demonstrated that there were no publication bias regarding the HRs of OS (*P* = 0.165) and PFS (*P* = 0.144).

## 4 DISCUSSION

In this study, we demonstrated that the pretreatment LDH was associated with PFS and OS in NSCLC patients treated with ICIs in the univariate and multivariate analysis. These results suggested that pretreatment LDH may serve as a potential predictor for ICIs in patients with non small cell lung cancer.

In the last decade, ICIs have brought a shift in the landscape of advanced‐stage cancer treatment. Despite of enormous success, not all patients achieve long‐lasting responses. Reliable predictive biomarkers remain to be found to identify patients who would benefit from ICIs. Systemic inflammatory status has been found closely correlated with worse prognosis in lung cancer.^7^, ^15^, ^16^ LDH as a classic inflammatory marker is correlated with tumor burden, which is released by rapidly growing tumors. Study has shown that a high LDH level may result in production of lactic acid and acidification of extracellular water space that contribute to an increased invasive ability of cancer cells.^17^ LDH has been found associated with poor prognosis when increased from 1 to 2.5 times ULN in patients treated with chemotherapy or targeted therapies.^6^, ^18^, ^19^ However, the effect of inflammatory status on benefit of ICI treatment in NSCLC patients is not well known yet, although some retrospective studies have shown that those with a high pretreatment of LDH level had a significantly shorter survival than those with normal LDH level.^9^, ^10^, ^11^, ^12^, ^13^, ^14^

The aim of this meta‐analysis is to investigate whether pretreatment LDH is correlated with clinical outcome of advanced NSCLC patients treated with ICIs. Previous studies have demonstrated that the level of serum LDH was significantly related to the extent of the tumor and poor prognosis in NSCLC patients.^18^, ^20^, ^21^ This meta‐analysis summarized the available evidence from 6 studies with 1136 cases. In univariate analysis, the pooled results suggested that elevated pretreatment LDH level was correlated with significant inferior PFS and OS and in multivariate analysis, the pooled results remained that a high baseline LDH level was strongly associated with poor PFS and OS. Multicenter retrospective study and a validation set demonstrated that lung immune prognostic index (LIPI), combining derived neutrophils/(leukocytes minus neutrophils) ratio (dNLR) and LDH are associated with worse outcome for ICI treatment in patients with advanced NSCLC, suggesting that LIPI might be a predictive tool on the prognosis of advanced NSCLC patients treated with ICIs.^10^ Therefore, an increased baseline level of serum LDH not only significantly correlates with clinical outcome of advanced NSCLC patients treated with ICIs, but also may have a predictive role on the prognosis of NSCLC patients treated with ICIs.

Above all, the understanding of LDH is still immature because of the lack of a uniform cut‐off value. Although baseline LDH level is correlated with the outcome of patients receiving ICIs, it remains uncertain what value of LDH is best to estimate the survival of patients with NSCLC. Furthermore, since LDH is a dynamic marker, when to measure LDH during a patient's treatment course is also unclear. The last but not the least, whether a single LDH determination or several over a time course is better at predicting survival in patients receiving ICIs has not been established.

Our study also has several limitations. First, the results of the meta‐analysis may jeopardize by the retrospective nature of included studies because of potential selection bias. Second, the number of studies included in the present meta‐analysis is relatively small, but the overall effect size is significant. Last but not least, the cut‐off value for LDH varied in these studies. The cut‐off values of LDH varied from 217 to 400 U/L. The level of LDH is influenced by the testing conditions, races, and age, which may be the cause of the difference in the cut‐off values of LDH. Even though, the difference in the cut‐off values may introduce bias to the results, the difference in the cut‐off values was minor.

## 5 CONCLUSION

In conclusion, this study demonstrates that a high pretreatment LDH level is statistically significantly associated with poor outcomes of NSCLC patients treated with ICIs. LDH is a potential useful predictive biomarker to select patients who can benefit from ICIs because of its convenient and non invasive nature. In the future, clinical trials are advocated to determine whether pretreatment LDH level could help stratify NSCLC patients who could benefit from ICIs.

## CONFLICT OF INTEREST

The authors declare no conflict of interest.
