# Supplementary material for: Pretreatment lactate dehydrogenase may predict outcome of advanced non small‐cell lung cancer patients treated with immune checkpoint inhibitors: A meta‐analysis
Source: Cancer Med. 2019 Mar 7;8(4):1467–73. doi: 10.1002/cam4.2024 (PMC6488146; doi:10.1002/cam4.2024)
Supplement: Supplementary file 2 [file CAM4-8-1467-s002.docx]

**FIGURE S1** Flow diagram of study inclusion and exclusion
